# Persistent Autoantibody-Production by Intermediates between Short-and Long-Lived Plasma Cells in Inflamed Lymph Nodes of Experimental Epidermolysis Bullosa Acquisita

**DOI:** 10.1371/journal.pone.0083631

**Published:** 2013-12-26

**Authors:** Benjamin Tiburzy, Martin Szyska, Hiroaki Iwata, Navina Chrobok, Upasana Kulkarni, Misa Hirose, Ralf J. Ludwig, Kathrin Kalies, Jürgen Westermann, David Wong, Rudolf Armin Manz

**Affiliations:** 1 Institute for Systemic Inflammation Research (ISEF), University of Lübeck, Lübeck, Germany; 2 Department of Dermatology, University of Lübeck, Lübeck, Germany; 3 Institute of Anatomy, University of Lübeck, Lübeck, Germany; University Hospital Jena, Germany

## Abstract

Autoantibodies are believed to be maintained by either the continuous generation of short-lived plasma cells in secondary lymphoid tissues or by long-lived plasma cells localized in bone marrow and spleen. Here, we show in a mouse model for the autoimmune blistering skin disease epidermolysis bullosa acquisita (EBA) that chronic autoantibody production can also be maintained in inflamed lymph nodes, by plasma cells exhibiting intermediate lifetimes. After EBA induction by immunization with a mCOL7c-GST-fusion protein, antigen-specific plasma cells and CD4 T cells were analyzed. Plasma cells were maintained for months in stable numbers in the draining lymph nodes, but not in spleen and bone marrow. In contrast, localization of mCOL7c-GST -specific CD4 T cells was not restricted to lymph nodes, indicating that availability of T cell help does not limit plasma cell localization to this site. BrdU-incorporation studies indicated that pathogenic mCOL7c- and non-pathogenic GST-specific plasma cells resemble intermediates between short-and long-lived plasma cells with half-lives of about 7 weeks. Immunization with mCOL7c-GST also yielded considerable numbers of plasma cells neither specific for mCOL7c- nor GST. These bystander-activated plasma cells exhibited much shorter half-lives and higher population turnover, suggesting that plasma cell lifetimes were only partly determined by the lymph node environment but also by the mode of activation. These results indicate that inflamed lymph nodes can harbor pathogenic plasma cells exhibiting distinct properties and hence may resemble a so far neglected site for chronic autoantibody production.

## Introduction

Serum autoantibodies are produced by either long- or short-lived plasma cells [1], exhibiting half-lives of a few days or several months, respectively [2]–[4]. While long-lived plasma cells are refractory to treatment with immunosuppressive treatment such as dexamethasone or cyclophosphamide [5], this treatment completely depletes short-lived plasma cells within one week [6]. In some patients suffering from autoimmune skin diseases, autoantibody production has been shown to be refractory to therapy, while in others autoantibodies may decline with various half-lives [7]. In treatment responders, autoantibodies decline within weeks up to three months [8], exhibiting half-live-times which are hardly explainable neither by autoantibody production through therapy susceptible short-lived plasma cells, nor through therapy resistant long-lived plasma cells [9]. Instead, it was suggested that the kinetics of autoantibody production during treatment of these patients may be explained by the destruction of niches supporting autoreactive plasma cells located within inflamed tissues. However, so far there is no experimental evidence supporting this idea.

Epidermolysis bullosa acquisita (EBA) is an organ-specific autoimmune disease clinically characterized by subepidermal blisters and immunologically by autoantibodies against type VII collagen (COL7), a main constituent of the anchoring fibrils at the dermal-epidermal junction [10], [11]. The pathogenic role of autoantibodies against type VII collagen has been demonstrated in humans and animal models [12]–[16]. Experimental EBA, which reproduces the immunopathological, histological, and clinical findings in patients with the inflammatory variant of EBA, is induced in susceptible mouse strains after a single immunization with mCOL7c-GST, a recombinant fusion protein consisting of a fragment of murine type VII collagen NC1 (mCOL7c) and a glutathione S-transferase (GST) tag as specified in the Material and methods section. Starting at week 2 after immunization, these experimental animals exhibit mCOL7c autoantibodies that persist for at least 12 weeks [14]. The effector phase of these antibodies, leading to tissue injury and blister formation, has been extensively investigated in patients and experimental animals both *ex vivo* and in vivo. In contrast, activation of autoreactive B and T lymphocytes and formation of plasma cells secreting these autoantibodies is poorly characterized [17]. Although it has been shown that T cells are involved in the generation of pathogenic autoantibodies in experimental EBA [18], a more detailed and complex understanding of the T cell and plasma cell response has so far not been obtained. In particular, it remained unclear if mCOL7c-specific T cells give help to mCOL7c-specific B cells. Alternatively, the mCOL7c-specific B cell response could be driven by GST-specific T cells, similar to what had been described for T cell dependent B cell responses against small hapten molecules such as (4-hydroxy-3-nitrophenyl)acetyl (NP). Although haptens such as NP are themselves not recognized by T cells, these antigens can elicit a T-dependent antibody response if coupled to carrier proteins [19], [20]. In such immune responses, T cell help is provided by carrier-specific T cells, allowing the generation of T-dependent B cell responses to antigens which as such do not activate a T cell response.

Here, we established methods for the identification of mCOL7c- and GST-specific T and plasma cells and applied these to investigate the lymphocyte responses in experimental EBA. These methods can be principally adopted to detect, quantify and analyze pathogenic lymphocytes specific for any self-antigen relevant in various autoimmune diseases in patients and mouse models. Our results suggest that T cell, but not B cell tolerance to mCOL7c may be crucial for preventing experimental EBA and that this disease is maintained by the continuous formation of plasma cells in lymph nodes harboring a COL7 specific plasma cell population with a half-life of several weeks. Production of autoantibodies in local sites of inflammation and their draining lymph nodes may explain the half-lives of autoantibodies during therapy observed in patients suffering from inflammatory autoimmune diseases, such as bullous skin diseases and vasculitis, among others.

## Results

### In Experimental EBA, COL7 Specific Plasma Cells are Found in Draining Lymph Nodes, but not in Spleen and Bone Marrow

**Figure 1:**
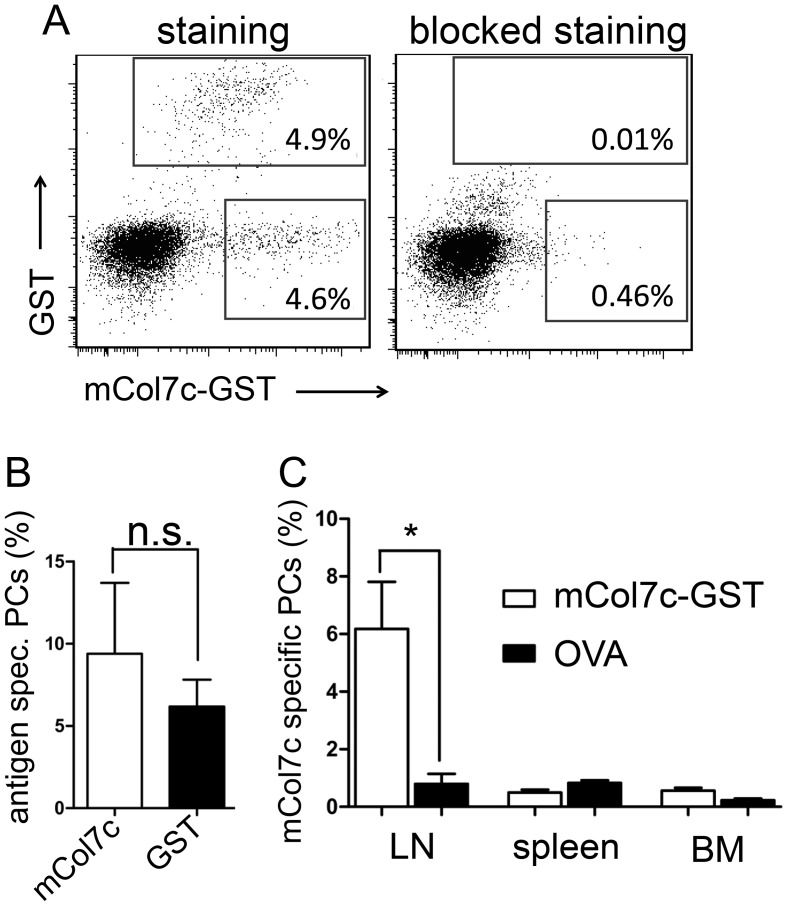
Identification of COL7-specific plasma cells (PCs). Cells from lymph nodes, spleen and bone marrow were stained as described in the Methods section (gating strategy: Figure S1). A, Left plot: population within the lower quadrant not binding to GST resembles cells specific for mCOL7c only. Right plot: Staining was blocked by pre-incubation with unlabeled antigens (mCOL7c-HIS and GST). B, Frequencies of plasma cells specific for mCOL7c or GST after mCOL7c-GST immunization, from the draining lymph nodes, 14 weeks after immunization. C, Frequencies of plasma cells specific for mCOL7c only, from the indicated organs of mice (n = 3–5) immunized either with mCOL7c-GST or OVA (as a control group), 14 weeks after immunization. Similar distribution of mCOL7c-specific plasma cells were found also weeks 3, 7 and 21 after immunization. Data are representative for three independent experiments.

Immunization with mCOL7c-GST fusion protein induces experimental EBA in mice of susceptible strains, such as SJL, starting at around 3–4 weeks after immunization [21]. In mice immunized a single time only, COL7-specific autoantibodies are induced that persist for at least 3 months [14], [21]. Here, we analyzed the plasma cell response underlying this antibody response. Plasma cells specific for mCOL7c and GST were identified by intracellular staining with antigen (Figure 1a and Figure S1), based on a modified version of methods that were used already earlier for the identification of plasma cells specific for other antigens, such as ovalbumin (OVA) or NP [22], [23]. Plasma cells specific for both antigens, GST and mCOL7c were found in the draining lymph nodes from all mice immunized with mCOL7c-GST in frequencies of about 5% among all plasma cells (Figure 1b). In contrast, lymph nodes of mice immunized with the control antigen OVA did not contain GST and mCOL7c plasma cells above the background, which could be blocked by excess of unlabeled antigen, of about 0.5% (Figure 1a/c). Additionally, immunization with the adjuvant (Titermax) alone did not induce mCOL7c- or GST-specific plasma cells (data not shown). In contrast to the draining lymph nodes, mCOL7c- or GST-specific plasma cells were not detectable in spleens and bone marrow (Figure 1c). These data show that the continuously activated lymph nodes are the major site for the production of COL7 specific autoantibodies in experimental EBA. This is in contrast to other autoimmune models, in which we and others have shown that autoantibodies are produced mainly in the spleen, bone marrow and/or inflamed tissues [5], [6], [24].

In contrast to plasma cells from spleen and bone marrow, lymph node plasma cells were not stained by CXCR3 or CXCR4 specific antibodies (Figure S2), indicating either lack of expression of these molecules or down-regulation due to extensive *in vivo* stimulation by their respective ligands in the environment of the active lymph node [25]. Particularly CXCR4 has been shown to be crucial for plasma cell homing to bone marrow and plasma cell distribution within the spleen [26]–[28]. In contrast, the expression of the CD62L was similar in all three tissues (Figure S2). Lack of expression or down-regulation of CXCR3 and CXCR4 surface expression in lymph node plasma cells from EBA mice may contribute to their limited homing capabilities, although this issue needs to be further investigated.

### mCOL7c and GST-specific Plasma Cell Populations Exhibit Similar Half-lives of Several Weeks

**Figure 2:**
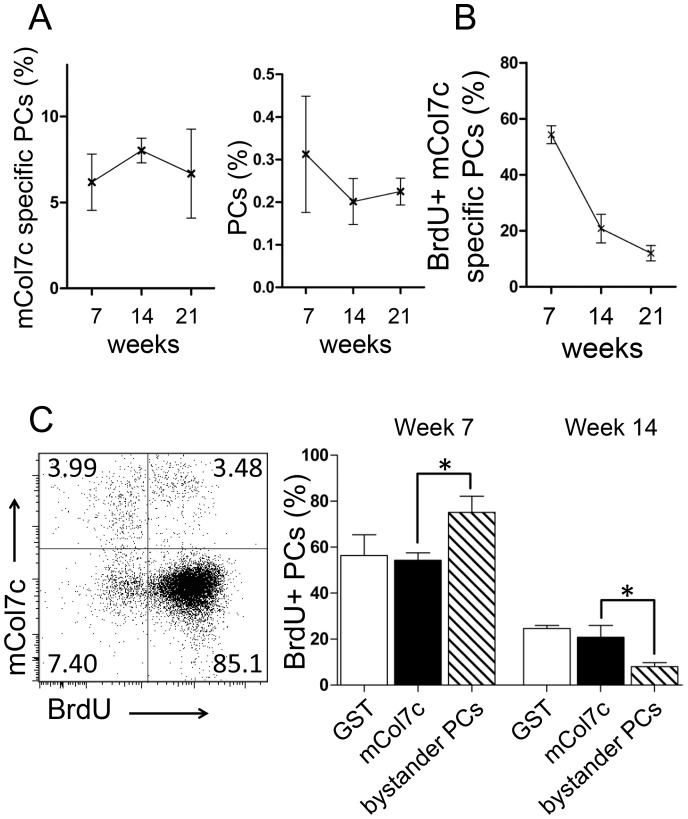
Turn-over of plasma cell populations. A, Frequencies of total plasma cells and mCOL7c-specific plasma cells among total plasma cells were determined at various time points after immunization as described in Fig. 2. B, from weeks 4 to 7 after immunization, mice were continuously fed with BrdU. Mice were analyzed directly after the pulse (week 7), as well as seven weeks (week 14) and 14 weeks (week 21) later. C, Left: GST-binding cells were excluded by electronic gating, allowing the identification of mCOL7c-specific plasma cells and plasma cells not binding to the immunizing antigens (bystander plasma cells neither specific for mCOL7c, nor GST). Right: Plasma cells specific for the indicated antigens were analyzed for BrdU incorporation directly after the feeding period at week 7, and 7 weeks later. Mean results (± SEM) are shown (n = 6). Data for week 7 and 14 are representative for two independent experiments.

Auto-antibodies can be maintained either by the continuous activation of B cells leading to the formation of short-lived plasma cells, or the persistence of long-lived plasma cells. Plasma cell formation is accompanied by cell proliferation as detected by the incorporation of the base analogue 5-bromo-2′-deoxyuridine (BrdU), while mature plasma cells can persist for several months without the need for proliferation [1], [9], [29], [30]. After the induction of the experimental EBA, for 21 weeks (the whole period of observation) the draining lymph nodes were drastically increased in size and contained about 100 million total cells, i.e. orders of magnitudes above the normal numbers (data not shown). BrdU was continuously fed via the drinking water over a 3-week-period from week 4 to 7. Frequencies of total plasma cells and mCOL7c-specific plasma cells in the draining lymph nodes remained constant between weeks 7, 14 and 21 after immunization with mCOL7c-GST (Figure 2a). The analysis of BrdU incorporation at week 7 indicated that within the 3-week-feeding-period, about 55% of the mCOL7c-specific plasma cells were newly generated (Figure 2b). Since BrdU incorporates efficiently in all newly formed plasma cells [6], this result also suggests that 45% of COL7-specific plasma cells had been formed before and persisted for at least 3 weeks without proliferation. At week 14, i.e. 7 weeks after BrdU feeding was stopped, still about 25% of mCOL7c- and GST-specific plasma cells were BrdU positive. At week 21, i.e. another 7 weeks later 10–15% BrdU+ plasma cells were detectable, suggesting that mCOL7c-specific plasma cells exhibit half-lives of approximately 7 weeks. Plasma cells specific for the non-self-antigen GST showed similar BrdU incorporation patterns than plasma cells specific for the self-antigen mCOL7c (Figure 2c). This is considerably longer than the few days of half-0,00life observed for short-lived plasma cells but much less than the minimum of 5 months half-life calculated for long-lived plasma cells [1]. Hence, autoreactive mCOL7c- specific and and non-self-reactive GST-specific plasma cells in the inflamed lymph nodes resemble intermediates between short-lived and long-lived plasma cells. The life-time of these immunization induced lymph node plasma cells is obviously not dependent on their specificity to self or non-self-antigens. Immunization also induced bystander plasma cells neither specific for mCOL7c-nor GST. In contrast to plasma cells induced by the immunizing antigens, these bystander-activated plasma cells are rather short-lived. From about 80% of BrdU labeled bystander plasma cells at week 7, only 10% remained BrdU+ till week 14 (Figure 2c). These results show that mCOL7c and GST-specific plasma cells exhibit considerably longer life-times than bystander-activated plasma cells formed during the same immune response within the same tissue. Together, these data indicate that plasma cell lifetimes are not only determined by the lymph node environment, but also by the mode of activation, although the self or non-self-nature of the antigen has no impact.

### Localization of mCOL7c-GST Specific CD4 T Cells is not Restricted to Lymph Nodes

**Figure 3:**
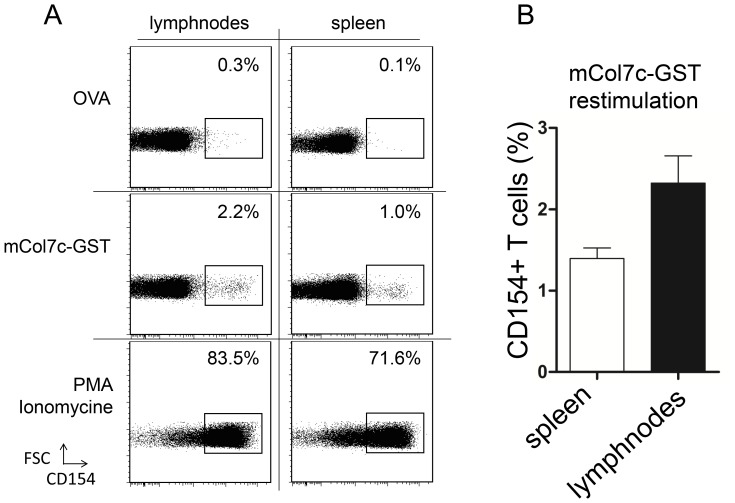
mCOL7c-GST specific CD4 T cells detected in spleen and draining lymph nodes after mCOL7c-GST immunization. Draining lymph node and spleen cells were harvested 7 weeks after immunization and restimulated with OVA (negative control), mCOL7c-GST or PMA and Ionomycin (positive control). A, representative flow cytometric analysis of restimulated CD 4 T cells. Cells were pre-gated according to CD4 expression. B, Frequencies of mCOL7c-specific cells obtained from draining lymph nodes and spleens 7 weeks after mCOL7c-GST immunization are shown (n = 8). Data are representative for more than three independent experiments.

The antibody response to COL7 in experimental EBA depends on T cell help [18]. In order to analyze the antigen-specific T cell response in our model, the frequencies of CD4 T cells specific for the mCOL7c-GST fusion protein were determined in SJL mice immunized with a fusion protein mCOL7c-GST by the rapid antigen-induced up-regulation of CD154, as described earlier [31], [32]. In the spleen and draining lymph nodes of all mice, CD4 T cells specific for the whole mCOL7c-GST fusion protein were detected at frequencies of about 1–2% (Figure 3a/b). The percentage of these cells is comparable to CD4 T cell frequencies specific for the immunizing antigens found after immunization with other antigens [32], [33]. Lymph nodes were massively expanded, but cell numbers within one lymph node did not exceed about 50% of cell numbers in spleen. Higher total cell numbers in spleen balanced for the lower frequencies of CD4 T cells specific for mCOL7c-GST, resulting in similar absolute numbers of these cells in both organs (Figure S3). These results show that in contrast to plasma cells, an expanded population of T cells specific for the immunizing antigen is also found outside the draining lymph nodes. Hence, the local restriction of the B cell response is not due to the lack of T cell help in other lymphoid tissues such as the spleen.

### GST-specific CD4 Cells Dominate the T Cell Response Against mCOL7c-GST

**Figure 4:**
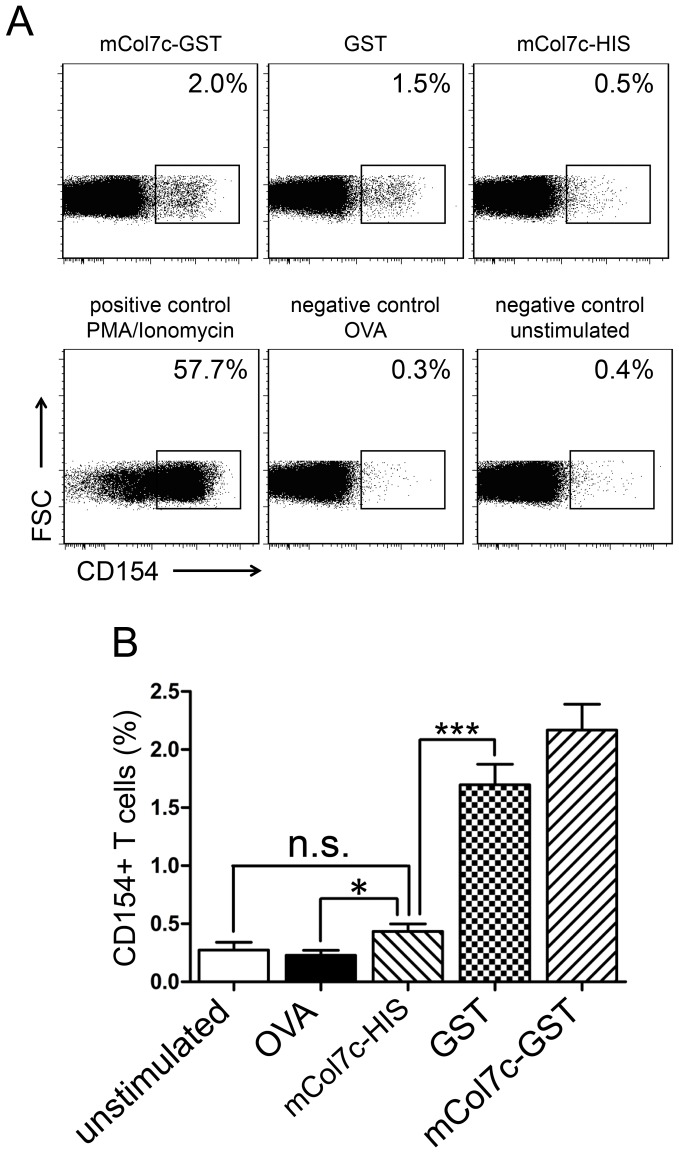
CD4 T cells specific for various antigens detected after mCOL7c-GST immunization. Draining lymph node cells were harvested 5 weeks after immunization and restimulated with no antigen, OVA, mCOL7c-HIS, GST or mCOL7c-GST, as indicated. T cells specific for the respective antigens were identified by electronic gating on CD4+ cells and the rapid up-regulation of CD154 [32]. A, representative flow cytometric analysis of cells stimulated with various antigens. Restimulation with PMA/Ionomycin was used as a positive control. B, Frequencies of antigen-specific cells obtained from draining lymph nodes 5 weeks after mCOL7c-GST immunization are shown (n = 6). Similar frequencies were determined at week 3 after immunization (data not shown). Data are representative for more than three independent experiments.

In order to address the question to which extend T cell tolerance to COL7 is broken in experimental EBA, the frequencies of CD4 T cells specific for the whole mCOL7c-GST fusion protein and specific for GST or mCOL7c only, were compared (Figure 4). Similar frequencies found in CD4 T cells specific for GST alone and for those specific for the whole mCOL7c-GST fusion protein were found, already suggesting that the great majority of CD4 T cells induced by immunization with the fusion protein are specific for GST. In accordance, frequencies of T cells reactive with mCOL7c (coupled to an irrelevant histidine(6)-tag (HIS)) were much lower and never significantly above both negative controls, i.e. unstimulated and OVA-restimulated cells (Figure 4a/b). When negative control values are subtracted, frequencies of mCOL7c-specific cells are not above 0.1% of all CD4 cells. These results show that the great majority of CD4 T cells expanded by immunization with mCOL7c-GST are specific for GST while mCOL7c-specific T cells are very rare or even absent. Hence, T cell tolerance to the autoantigen mCOL7c seems to be relatively intact even after induction of EBA by immunization with mCOL7c-GST.

### Protein or Peptide Tags Coupled to the Immunizing mCOL7c Peptide Modulate mCOL7c-specific Plasma Cell Responses and Induction of EBA

**Figure 5:**
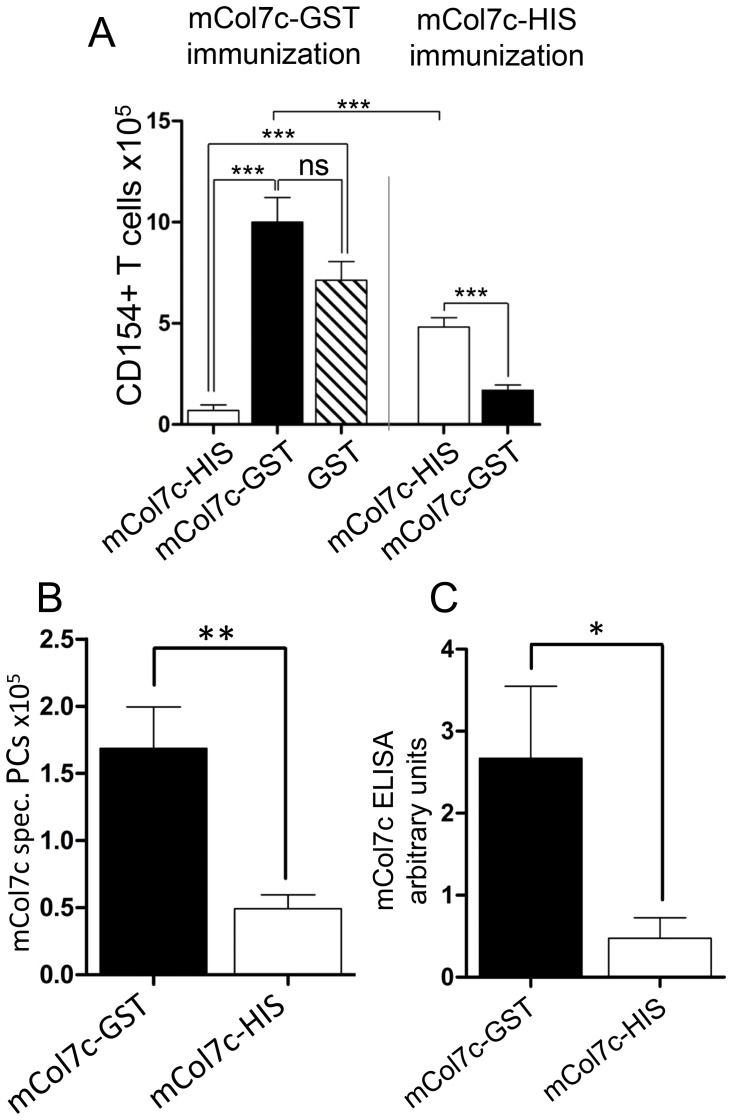
T cells, plasma cells and antibodies specific for immunizing antigens. Mice were immunized with either mCOL7c-GST or mCOL7c-HIS. A, CD4 T cells from draining lymph nodes specific for mCOL7c-HIS, mCOL7c-GST and GST were determined as CD154+ described in Fig. 1. Average background values of negative controls (unstimulated and OVA stimulated cells) were subtracted in all samples. Examples show T cell numbers at week 3 after immunization (n = 6). B, In the same experiment, the numbers of plasma cells specific for mCOL7c were determined as described in Fig. 2 (n = 6). C, mCOL7c-specific serum antibodies 8 weeks after immunization with mCOL7c-GST or mCOL7c-HIS determined by ELISA (n = 3–4). Data are representative for two independent experiments.

As shown above, immunization with mCOL7c-GST induces plasma cells specific for both antigens mCOL7c and GST. In contrast, the T cell response is dominated by a response against GST while mCOL7c-specific CD4 T cells are hardly detectable. These results may indicate that GST- specific CD4 T cells may drive the mCOL7c-specific plasma cell response, similar to carrier-protein-specific T cells that support B cell responses to small haptens [19], [20]. To test this idea further, mice were immunized with mCOL7c-GST or mCOL7c-HIS. In mice immunized with mCOL7c-HIS, lower numbers of T cells specific for the immunizing antigens are found compared to immunization with mCOL7c-GST. Moreover, most of these T cells are specific for a peptide containing the HIS-tag, but not for mCOL7c (Figure 5a). Accordingly, mCOL7c-HIS immunization results in only about 25% of the mCOL7c-specific plasma cells as compared to the immunization with mCOL7c-GST (Figure 5b), mCOL7c-specific antibodies were reduced even to a higher extend (Figure 5c). Accordingly, EBA skin disease is induced after immunization with mCOL7c-GST, as published before [14], but not with mCOL7c-His or mCOL7c alone (Figure S4 and Table S1). These results show that the tag coupled to the immunizing mCOL7c modulates mCOL7c-specific plasma cell responses and the induction of EBA. Also, they suggest that T cell tolerance to mCOL7c is kept intact at least to the extent that T cell help for B cells is limited and disease induction is prevented in the absence of an immunogenic protein tag presented on mCOL7c reactive B cells. In such a scenario, mCOL7c reactive B cells take up the whole fusion protein, present all its peptides on MHCII molecules and can receive help from tag-specific T cells.

## Discussion

Here, we established methods suitable for the complementary analysis of antigen-specific plasma cell- and T cell responses in a mouse model off EBA. Interestingly, and in contrast to several other immunization-induced chronic inflammatory disease models [5], [6], [24], our results show that the COL7-specific plasma cell response in the experimental model of EBA is maintained for several months in the draining lymph nodes. Both, prolonged survival and continuous new formation of plasma cells contributed to this extended antibody response, with an overall plasma cell lifetime in-between short-and long-lived cells. Interestingly, formation and survival of mCOL7c-specific plasma cells was similar to that observed for plasma cells specific for GST. Despite the ubiquitous presence of the self-antigen COL7, plasma cells specific for both antigens were only found in draining lymph nodes. This observation suggests that the generation of plasma cells is restricted to these organs. Localization of plasma cells within secondary lymphoid organs is typical for a single immunization with non-self-antigens. However, usually, these responses are transient [1], [34]. Only after repeated antigenic stimulation, plasma cell responses move to the bone marrow where they become long-lived. Based on these criteria, the persistent autoreactive plasma cell response within the lymph nodes observed in this study neither resembles a typical primary nor a typical secondary response. To better understand this phenomenon, we performed a complementary analysis of the mCOL7c- and GST-specific T cells. After immunization with mCOL7c-GST, CD4 T cells specific for mCOL7c were nearly undetectable while T cells specific for GST were found in much higher numbers, but comparable to those described after immunization with other protein antigens [33]. These data suggest that the mCOL7c-specific plasma cell response is driven by T cells specific for GST. One may speculate that B cells specific for mCOL7c bind and internalize the entire mCOL7c-GST fusion protein, present GST derived peptides and receive help by GST-specific T cells.

In contrast to the self-antigen COL7, the foreign antigen GST is present after immunization only locally and most likely activates GST-specific T cells only in the draining lymph nodes, despite their also occurring presence in the spleen. Hence, explaining the presence of mCOL7c-specific plasma cells in draining lymph nodes but not in other tissues. In addition, the adjuvant used in experimental EBA leads to a chronic inflammation of draining lymph nodes (data not shown) and we speculate that the inflammatory environment also contributes to the retention of plasma cells. This idea is supported by the observation that plasma cells formed in the course of inflammatory responses express chemokine receptors mediating homing to sites of inflammation [28], [35], [36].

Our data suggest that T cell tolerance for COL7 is intact in the experimental model of EBA. In accordance with this idea, immunization with mCOL7c coupled to a HIS-tag again led to a very low T cell response against mCOL7c. After immunization with mCOL7c-HIS, the T response against the HIS-tagged mCOL7c was higher than against the GST-tagged mCOL7c, but still lower than the response against GST induced by mCOL7c-GST immunization. Likewise, mCOL7c-specific plasma cell numbers and antibody levels were reduced after immunization with mCOL7c-HIS compared to mCOL7c-GST, clearly demonstrating that mCOL7c-specific antibody production is partly determined by the immunogenicity of the tag coupled to mCOL7c. Moreover, the failure to induce disease after immunization with mCOL7c-HIS also suggests that the tag is relevant for pathogenicity. While B cells recognize whole protein antigens, T cells are specific for small peptide antigens, resembling certain epitopes of the protein. During active disease in EBA patients, T cell tolerance against some epitopes of COL7 is likely to be broken. Alternatively, T cell help may be provided by T cells specific for epitopes of infectious agents exhibiting similarities with COL7 epitopes or by molecular mimicry as postulated for other autoimmune diseases [37]. It would be interesting to adopt the methods described here to analyze human T cells specific for COL7 epitopes, to identify those epitopes to which tolerance is broken. This would allow investigating whether these epitopes show similarities to common infectious agents.

After systemic immunization/vaccination, memory antibodies are maintained by long-lived plasma cells in the bone marrow [1]. Maintained autoantibody production has also been described in spleen and inflamed kidneys, as shown in murine lupus [6], [38]. In contrast to the production of protective antibodies, autoantibodies can be maintained by both the continuous formation of short-lived plasma cells and/or the persistence of long-lived plasma cells. While the former exhibit half-lives of a few days, the latter persist for many months at least [2], [4], [6]. Here we describe a chronic autoreactive plasma cell response in inflamed lymph nodes. Hence, it could be speculated that the localization of autoreactive plasma cells differs between various diseases, and possibly even between individual patients. This could be important because it had been shown that plasma cell localization determines their susceptibility to therapeutic treatment [5]. Therefore, after identification of diagnostic correlates for autoreactive plasma cell localization, these could become important tools for selection of appropriate therapeutic options. While in our model the immunizing antigen is localized by subcutaneous immunization to a certain site, the presence of type VII collagen is naturally restricted to skin and a few other tissues. Based on the observations in our model it seems possible that the local availability of COL7 restricts autoantibody production to skin draining lymph nodes also in EBA patients.

In patients with autoimmune bullous diseases that respond well to therapy, autoantibodies typically decline slowly within 8–12 weeks [8], [39]–[41]. Production of autoantibodies by an intermediate cell type between short- and long-lived plasma cells, located in inflamed lymph nodes or tissues, provides an explanation for this observed kinetics of decline of autoantibodies during therapy, which is otherwise hardly explainable neither by autoantibody production through therapy susceptible short-lived plasma cells, nor through therapy resistant long-lived plasma cells.

## Materials and Methods

### Mice

8-week-old SJL mice were purchased from Charles River Laboratories (Sulzfeld, Germany). Experiments were performed at the animal facility of the University of Lübeck. All animal experiments were approved by the respective local Committee on the Ethics of Animal Experiments of the state Schleswig-Holstein (Ministerium für Landwirtschaft, Umwelt und ländliche Räume des Landes Schleswig Holstein), proposal „Entstehung des autoreaktiven Antikörpergedächtnis gegen Typ VII Kollagen bei der Epidermolysis Bullosa Acquisita (EBA)“, permit number V3172241.73/10. All animal experiments were performed by certified personnel and all efforts were made to minimize suffering.

### Production of Autoantigen and Coupling to Fluorochromes

The recombinant fragment of murine type VII collagen (mCOL7c; amino acids 757–967) was prepared as described previously [13]. Recombinant tagged fragments mCOL7c-GST and HIS-tagged mCOL7c as well as GST were produced using a prokaryotic expression system and purified by glutathione and metallochelate affinity chromatography, respectively. The proteins were coupled to Alexa Flour 700 and Alexa Flour 488 (Life Technologies GmbH, Darmstadt, Germany) according to protocols provided by the manufacturer.

### Induction and Evaluation of EBA

Experimental EBA was induced in mice by active immunization, as described previously [14], [17]. Briefly, mice were injected subcutaneously in the hind footpads with a single injection of 60 µL of emulsion containing 60 µg of mCOL7c-GST or mCOL7c-HIS in TiterMax (Alexis Biochemicals, Lörrach, Germany). Disease severity was determined by examination for erythema, blisters, erosions, and crusts as described earlier [13] and expressed as % affected body surface area.

### Flow Cytometry and Antibodies

Total cell numbers of single cell suspensions from draining lymph nodes (popliteal and inguinal) and spleens were determined by using a HEMAVET 950 cell counter (Drew Scientific Inc., Dallas, USA). Cells were stained with PE-conjugated anti-mouse Syndecan-1 (CD138, clone 281-2, BD Biosciences, Pharmingen), Alexa-405 (Life Technologies GmbH, Darmstadt, Germany) coupled anti-mouse B220 (clone RA3.B2, in house production), Alexa-eFluor450 coupled anti-mouse CD4 (clone GK1.5, eBiosciences, Frankfurt, Germany), PE coupled anti-mouse CD154 (clone MR1, BioLegend, Fell, Germany) and fluorochrome coupled antigens. Cells were fixed prior to intracellular staining using Cytofix/Cytoperm (BD Biosciences, Pharmingen). Samples were analyzed on a BD Biosciences LSRII flow cytometer, and the resulting data were evaluated using FlowJo software (TreeStar Inc., Ashland, USA). Due to the rarity of the analyzed events, at least one million cells were recorded for each sample.

### Detection of Antigen-specific T Cells

Antigen-specific T cells were detected by the CD40L (CD154) expression method as described previously [32]. *Ex vivo* restimulation of single cell suspensions from draining lymph nodes (popliteal and inguinal) or spleen was performed with 100 µg of mCOL7c-GST, HIS-tagged mCOL7c or GST in RPMI1640 Medium (Life Technologies GmbH, Darmstadt, Germany) in 48well flat bottom plates. As a negative control, 100 µg of Ovalbumin or 100 µl PBS was used for restimulation, high level CD154 expression as positive control was achieved by PMA (Sigma Aldrich, Munich, Germany) and Ionomycin (Merck Biosciences, Schwalbach, Germany) stimulation. To detect the expression of CD154 of the CD4 T cells, cells where restimulated in the presence of Monensin (eBiosciences, Frankfurt, Germany), followed by fixation and intracellular staining.

### Detection of Antigen-specific Plasma Cells

Single cell suspensions from draining lymph nodes, spleen and bone marrow were stained for B220 and CD138, fixed with Cytofix/Cytoperm (BD Biosciences, Pharmingen) and subsequently stained intracellularly with fluorochrome labeled GST and mCOL7c-GST, similar to what was described for staining of plasma cells specific for other antigens [23].

### ELISA

Serum antibodies were detected as described before [14], each well of a 96well plate was coated with 500ng of recombinant HIS-tagged mCOL7c in 0.1M bicarbonate buffer (pH 9.6). After blocking, wells were incubated with a 150-fold dilution of mouse sera for 60 min. Bound antibodies were detected using a 500-fold dilution of a biotinylated goat anti-mouse IgG antibody (Southern Biotech, Birmingham, USA), a 3000-fold dilution of streptavidin coupled alkaline phosphatase (Roche Diagnostics GmbH, Mannheim, Germany) and ALP (Roche Diagnostics GmbH, Mannheim, Germany). Readout was performed on a FLUOstar Omega Microplate Reader (BMG LABTECH GmbH, Ortenberg, Germany); ODs were analyzed by a (5-PL) Non-Linear Regression Curve-Fitting Model.

### Statistics

Statistical calculations were performed using GraphPad Prism (GraphPad Software, La Jolla, USA). Significances were calculated by students T test and expressed as mean ± SEM. *P<0.05, **P<0.01, ***P<0.001.

## Supporting Information

Figure S1 **Gating strategy for the identification of antigen-specific plasma cells in a flow cytometer.** Life cells were stained for B220 and CD138, fixed and subsequently stained intracellular with GST and mCOL7c-GST. During acquisition, debris was excluded using a forward/sideward scatter gate. B220 intermediate/CD138 positive cells were selected, followed and smaller cells and artifacts were eliminated by a second forward/sides scatter gate. Within the remaining population of total plasma cells, those specific for the immunizing antigens GST and mCOL7c were detected within two distinct populations of cells binding to mCOL7c-GST alone (lower left plot, lower quadrant), or mCOL7c-GST and GST (lower left plot, upper quadrant).
(TIF)

Figure S2 **Flow cytometric analysis of migration associated molecules.** Cells were isolated from lymph nodes, spleens and bone marrow of EBA mice 14 weeks after immunization, stained for the markers indicated and analyzed by flow cytometry. Histogram overlays show the expression of the indicated markers for cells from lymph nodes (red), spleens (blue), bone marrow (orange) and an internal negative control from spleen (black). Data are representative for 5 individual mice separately analyzed in one experiment.
(TIF)

Figure S3 **Absolute numbers of mCOL7c-GST specific CD4 T cells.** Single cell suspensions were prepared from lymph nodes and spleens 7 weeks after mCOL7c-GST immunization. Total cell numbers were quantified using a cell counter (HEMAVET 950). Frequencies of mCOL7c-GST specific T cells were determined by flow cytometry as described in the Material and Methods section. Absolute numbers of mCOL7c-GST specific T cells were calculated on the basis of their frequencies and the total numbers per organ (n = 8). Data are representative for more than three independent experiments.
(TIF)

Figure S4 **Representative pictures of clinical scoring.** Mice were immunized with either mCOL7c-GST, mCOL7c-HIS or untagged mCOL7c, as indicated. Pictures were taken 8 weeks after immunization. Examples shown are representative for 3–4 mice per group, as shown in Table S1.
(TIF)

Table S1 **Mice were immunized with either mCOL7c-GST, mCOL7c-HIS or untagged mCOL7c and scored for EBA affected body surface area as described earlier** [**14**] **.** Representative pictures are shown in Figure S4.
(DOC)
