# Supplementary figures and images for: Persistent Autoantibody-Production by Intermediates between Short-and Long-Lived Plasma Cells in Inflamed Lymph Nodes of Experimental Epidermolysis Bullosa Acquisita
Source: PLoS One. 2013 Dec 26;8(12):e83631. doi: 10.1371/journal.pone.0083631 (PMC3873383; doi:10.1371/journal.pone.0083631)

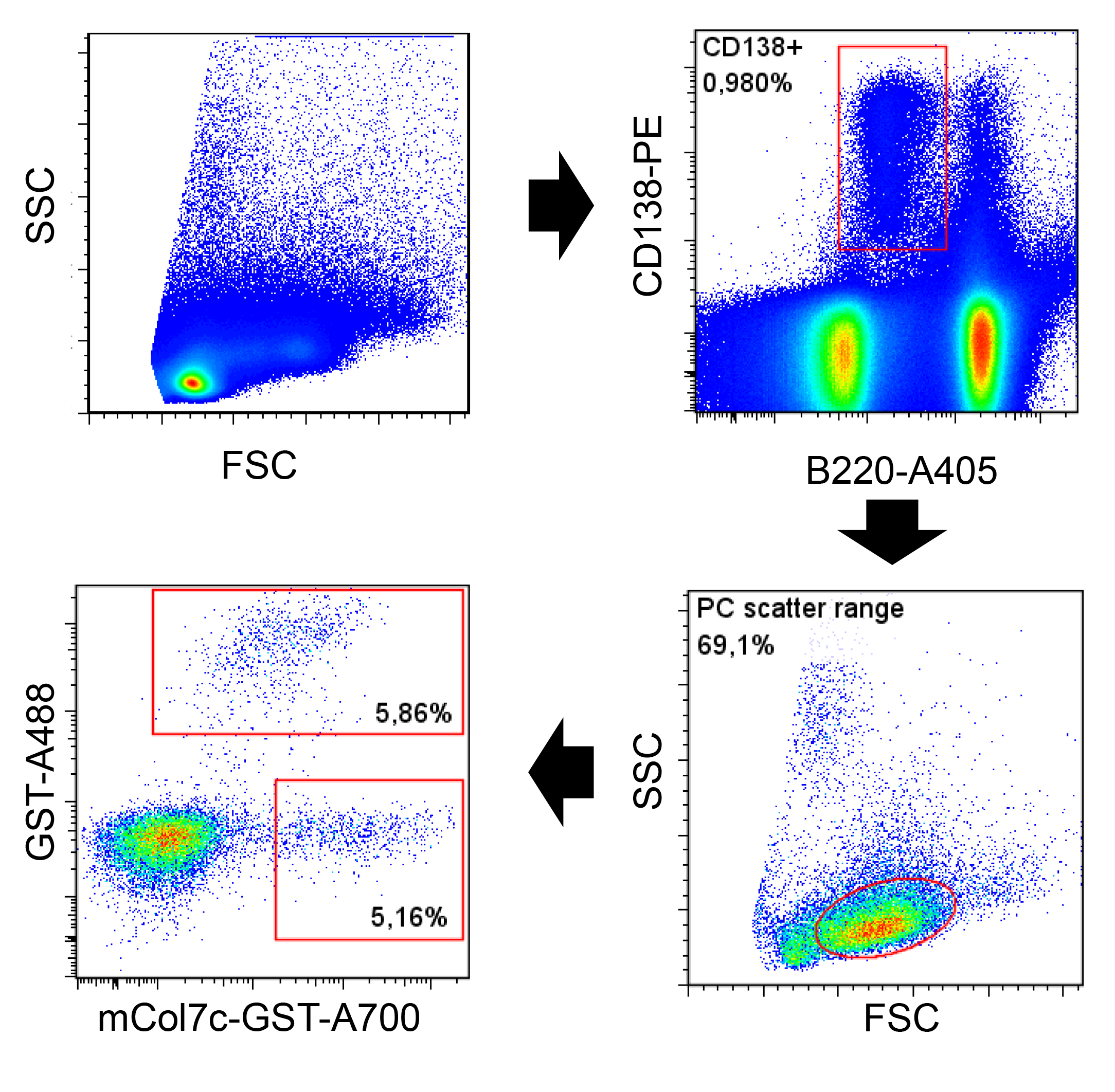

Supplement: Figure S1 — Gating strategy for the identification of antigen-specific plasma cells in a flow cytometer. Life cells were stained for B220 and CD138, fixed and subsequently stained intracellular with GST and mCOL7c-GST. During acquisition, debris was excluded using a forward/sideward scatter gate. B220 intermediate/CD138 positive cells were selected, followed and smaller cells and artifacts were eliminated by a second forward/sides scatter gate. Within the remaining population of total plasma cells, those specific for the immunizing antigens GST and mCOL7c were detected within two distinct populations of cells binding to mCOL7c-GST alone (lower left plot, lower quadrant), or mCOL7c-GST and GST (lower left plot, upper quadrant). (TIF) [file pone.0083631.s001.tif]

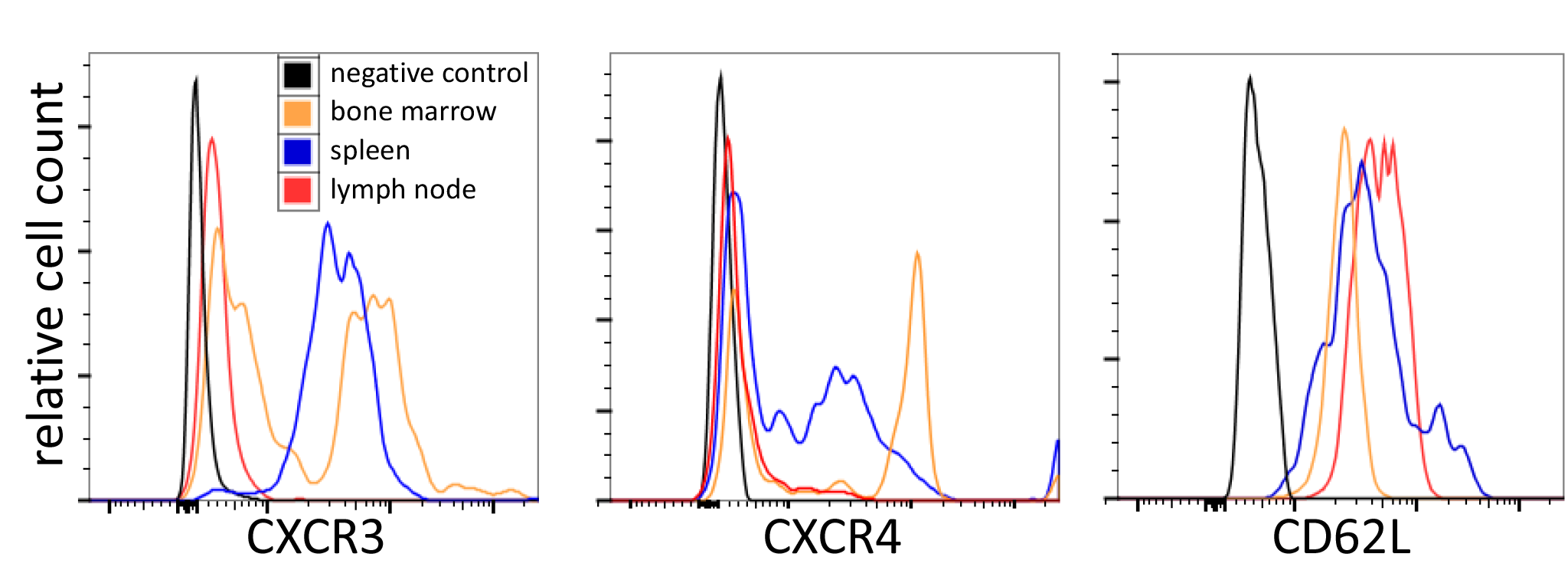

Supplement: Figure S2 — Flow cytometric analysis of migration associated molecules. Cells were isolated from lymph nodes, spleens and bone marrow of EBA mice 14 weeks after immunization, stained for the markers indicated and analyzed by flow cytometry. Histogram overlays show the expression of the indicated markers for cells from lymph nodes (red), spleens (blue), bone marrow (orange) and an internal negative control from spleen (black). Data are representative for 5 individual mice separately analyzed in one experiment. (TIF) [file pone.0083631.s002.tif]

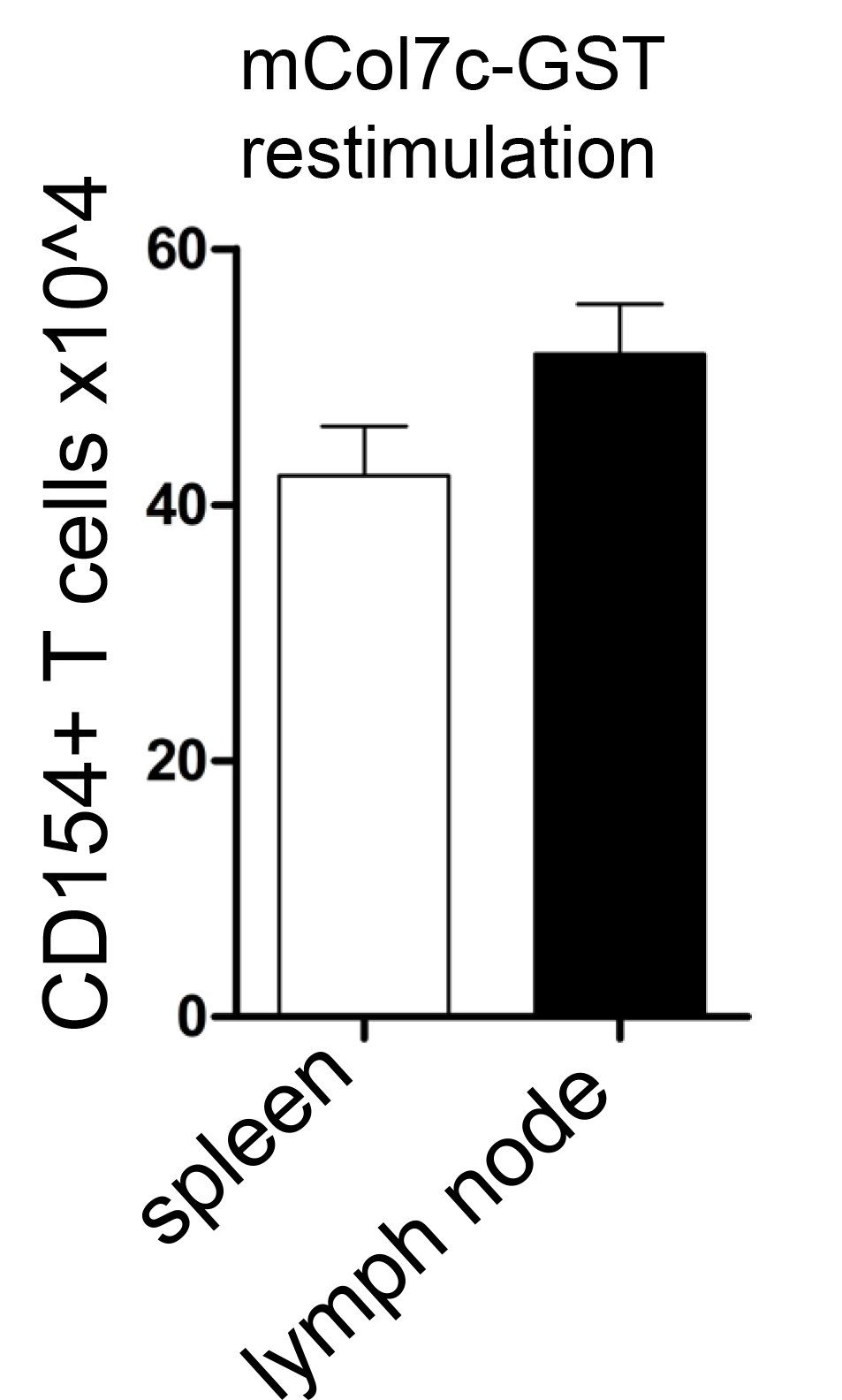

Supplement: Figure S3 — Absolute numbers of mCOL7c-GST specific CD4 T cells. Single cell suspensions were prepared from lymph nodes and spleens 7 weeks after mCOL7c-GST immunization. Total cell numbers were quantified using a cell counter (HEMAVET 950). Frequencies of mCOL7c-GST specific T cells were determined by flow cytometry as described in the Material and Methods section. Absolute numbers of mCOL7c-GST specific T cells were calculated on the basis of their frequencies and the total numbers per organ (n = 8). Data are representative for more than three independent experiments. (TIF) [file pone.0083631.s003.tif]

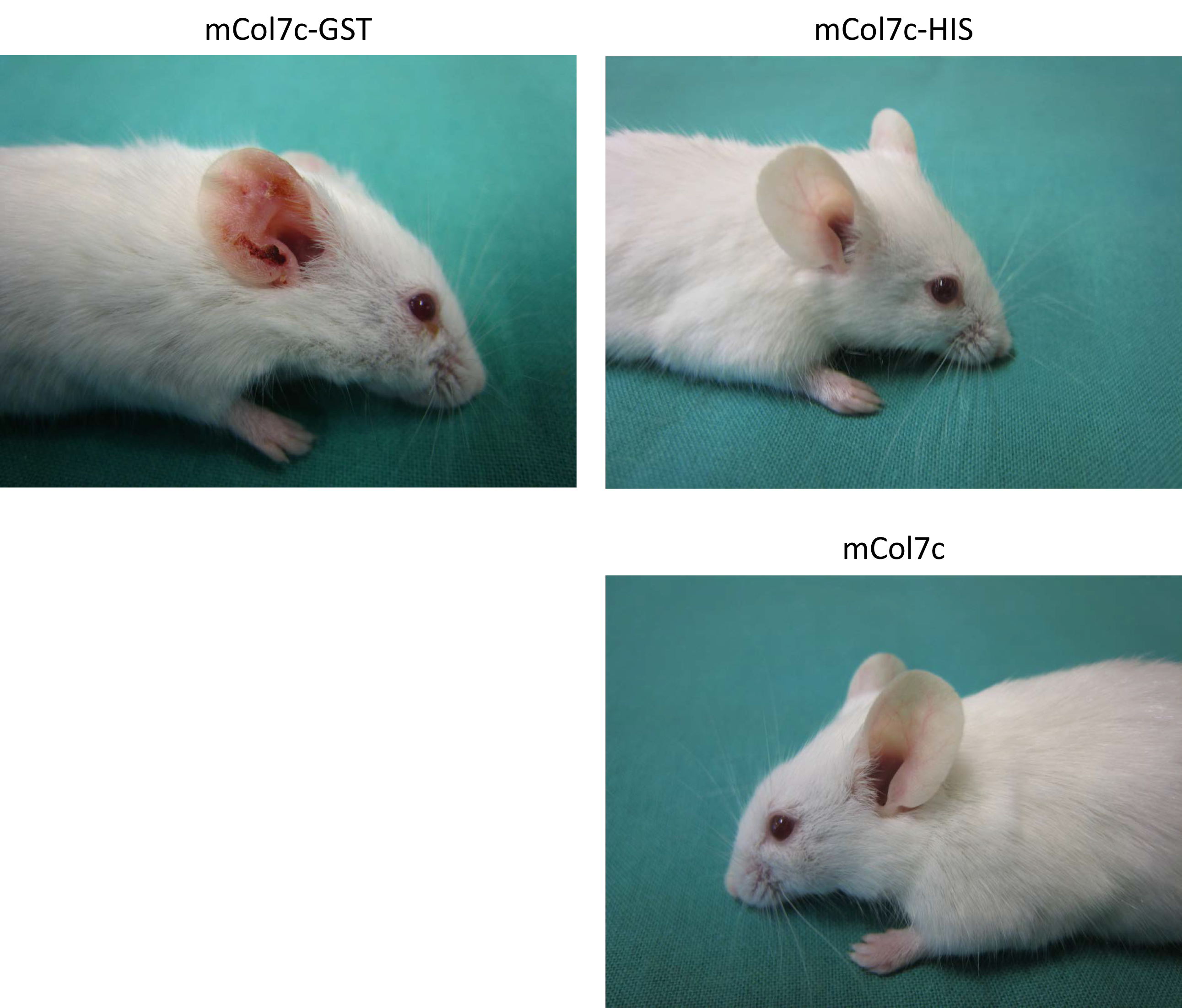

Supplement: Figure S4 — Representative pictures of clinical scoring. Mice were immunized with either mCOL7c-GST, mCOL7c-HIS or untagged mCOL7c, as indicated. Pictures were taken 8 weeks after immunization. Examples shown are representative for 3–4 mice per group, as shown in Table S1. (TIF) [file pone.0083631.s004.tif]
