# Supplementary material for: Persistent Autoantibody-Production by Intermediates between Short-and Long-Lived Plasma Cells in Inflamed Lymph Nodes of Experimental Epidermolysis Bullosa Acquisita
Source: PLoS One. 2013 Dec 26;8(12):e83631. doi: 10.1371/journal.pone.0083631 (PMC3873383; doi:10.1371/journal.pone.0083631)
Supplement: Table S1 — Mice were immunized with either mCOL7c-GST, mCOL7c-HIS or untagged mCOL7c and scored for EBA affected body surface area as described earlier [14] . Representative pictures are shown in Figure S4. (DOC) [file pone.0083631.s005.doc]

|  | **clinical scoring (% body surface)** | | |
| --- | --- | --- | --- |
| **mouse** | **mCol7c-GST** | **mCol7c-HIS** | **mCol7c** |
| **1** | 0,2 | 0 | 0 |
| **2** | 1,4 | 0 | 0 |
| **3** | 1,3 | 0 | 0 |
| **4** |  | 0 | 0 |
